# Factors influencing the outcomes of contrast-enhanced ultrasound in the detection of sentinel lymph nodes in breast cancer

**DOI:** 10.3389/fonc.2025.1662625

**Published:** 2025-10-15

**Authors:** Jun Luo, Yunhao Luo, Wenbin Cao, Liting Feng, Chaonan Li, Yuyan Liu, Dayan Huang, Cheng Guan, Qiang Lu

**Affiliations:** ^1^ Department of Ultrasound, West China Hospital of Sichuan University, Chengdu, China; ^2^ Department of Ultrasound, Sichuan Provincial People’s Hospital, University of Electronic Science and Technology of China, Chengdu, China; ^3^ School of Medical and Life Sciences, Chengdu University of Traditional Chinese Medicine, Chengdu, China; ^4^ Ultrasonic Medical Department, Hospital of Chengdu University of Traditional Chinese Medicine, Chengdu, China; ^5^ Department of Ultrasound, Chengdu Second People’s Hospital, Chengdu, China

**Keywords:** contrast-enhanced ultrasound, breast cancer, sentinel lymph node, influencing factors, logistic regression

## Abstract

**Objective:**

To analyze the influencing factors on the failure of sentinel lymph node (SLN) imaging in breast cancer by percutaneous injection contrast-enhanced ultrasound (CEUS).

**Methods:**

Clinical data including basic characteristics, medical history, ultrasound technical parameters, and laboratory findings were collected from the patients, and the pathological results of sentinel lymph node biopsy (SLNB) were used as the gold standard for all patients. Logistic regression was used to analyze all influencing factors. The performance of the model was assessed using the area under the receiver operating characteristic curve (AUC), P<0.05 was statistically significant. Nomogram was developed for the assessment of risk prediction.

**Results:**

A total of 1356 patients were included, of which 92.48% (1254/1356) had successful imaging and 7.52% (102/1356) had failed imaging. Logistic regression analysis showed that the quadrant location of tumor [(OR = 1.54, 95%CI: 1.01, 2.36)], the history of previous breast surgery [(OR = 3.05, 95%CI: 1.62, 5.72)], the metastasis of SLN [(OR = 2.68, 95%CI: 1.72, 4.15)], the history of neoadjuvant therapy [(OR = 2.90, 95% CI: 1.57, 5.37)], and the clinical prognostic stage [(OR = 3.50, 95% CI: 1.70, 7.22)] were the independent influencing factors of CEUS failure, with a statistically significant difference (P<0.05). The AUC of its predictive model in the training set was 0.72 (95% CI: 0.65-0.78), and the AUC of the predictive model in the validation set was 0.71 (95% CI: 0.61-0.81).

**Conclusions:**

The quadrant location of tumor, the history of previous breast surgery, the metastasis of SLN, the clinical prognostic stage and the history of previous neoadjuvant therapy were the independent influencing factors for the failure of CEUS for SLN imaging in breast cancer.

## 1 Introduction

Breast cancer is the most prevalent malignant tumor worldwide and is the second leading cause of death from malignant tumors in women (1). For patients with early-stage breast cancer, sentinel lymph node biopsy (SLNB) is the standard treatment (2). Sentinel lymph node (SLN) is the first lymph node for lymphatic drainage and the first station for axillary lymph node (ALN) metastasis in breast cancer. It is of great clinical significance to accurately determine whether SLN metastasis exists before the surgery (2). Percutaneous contrast-enhanced ultrasound (CEUS) has great potential for SLN imaging due to its real-time, radiation-free, and high-resolution advantages (3, 4). After the ultrasound contrast agent has been injected into the areola area and absorbed by the lymphatic system, the operator can track and locate the SLN along the lymphatic vessels, thereby achieving preoperative localization of the SLN and even determining whether there is metastasis. Previous multicenter study (3, 5) and our studies (6, 7) have demonstrated that the use of CEUS can accurately identify SLN in breast cancer patients, but there were still 3.63% ~ 6.52% of patients in whom CEUS failed for imaging—defined as failure to clearly visualize the SLN (6, 7)—which poses a challenge to clinical practice. Currently, no study has been reported to analyze the failure factors of CEUS for SLN imaging in breast cancer. Therefore, our study aims to use multifactor analysis to explore the influencing factors of CEUS failure to guide clinical decision making.

## 2 Materials and methods

### 2.1 Basic information of patients

This study retrospectively included patients who underwent CEUS for breast cancer SLN imaging from June 2017 to June 2024 at Sichuan Provincial People's Hospital, China. The study was approved by the Ethics Committee of the Sichuan Provincial People's Hospital, China, and informed consent was waived (Approval No. 2022.263). Clinical data including age, medical history (surgical history referred specifically to a history of the outer upper quadrant surgery on the breast.), ultrasound technical parameters, and laboratory findings were collected from the patients, and the pathological diagnosis after SLNB surgery as the gold standard for all patients. The classification of clinical prognostic staging follows the 8th edition of the AJCC Breast Cancer Treatment Guidelines (8).

Inclusion criteria: 1. Female, age ≥18 years; 2. Pathologically confirmed breast malignancy, T1–3 and ductal carcinoma *in situ* (DCIS) scheduled for surgical treatment (total mastectomy or breast-conserving surgery); 3. Preoperative clinical diagnosis of ALN negativity, defined as no evidence of ALN metastasis via clinical examination (no palpable lymphadenopathy with hard texture, fixed, or matting), imaging modalities (ultrasound and magnetic resonance imaging when clinically indicated, with no ALN showing malignant characteristics like irregular shape, interrupted cortex, or internal calcification), and histopathological confirmation (only performed for ALNs with suspicious imaging findings, with no metastasis confirmed); 4. Completion of percutaneous injection of CEUS for SLN imaging. 5. Underwent standard SLNB.

Exclusion criteria: 1. ALN metastasis confirmed by pathological examination (core needle biopsy or fine-needle aspiration cytology) before CEUS; 2. Inflammatory breast cancer; 3. Pregnancy; 4. Poor cooperation with the CEUS examination resulting in image quality not meet diagnostic criteria.

### 2.2 CEUS examination

**Figure 1:**
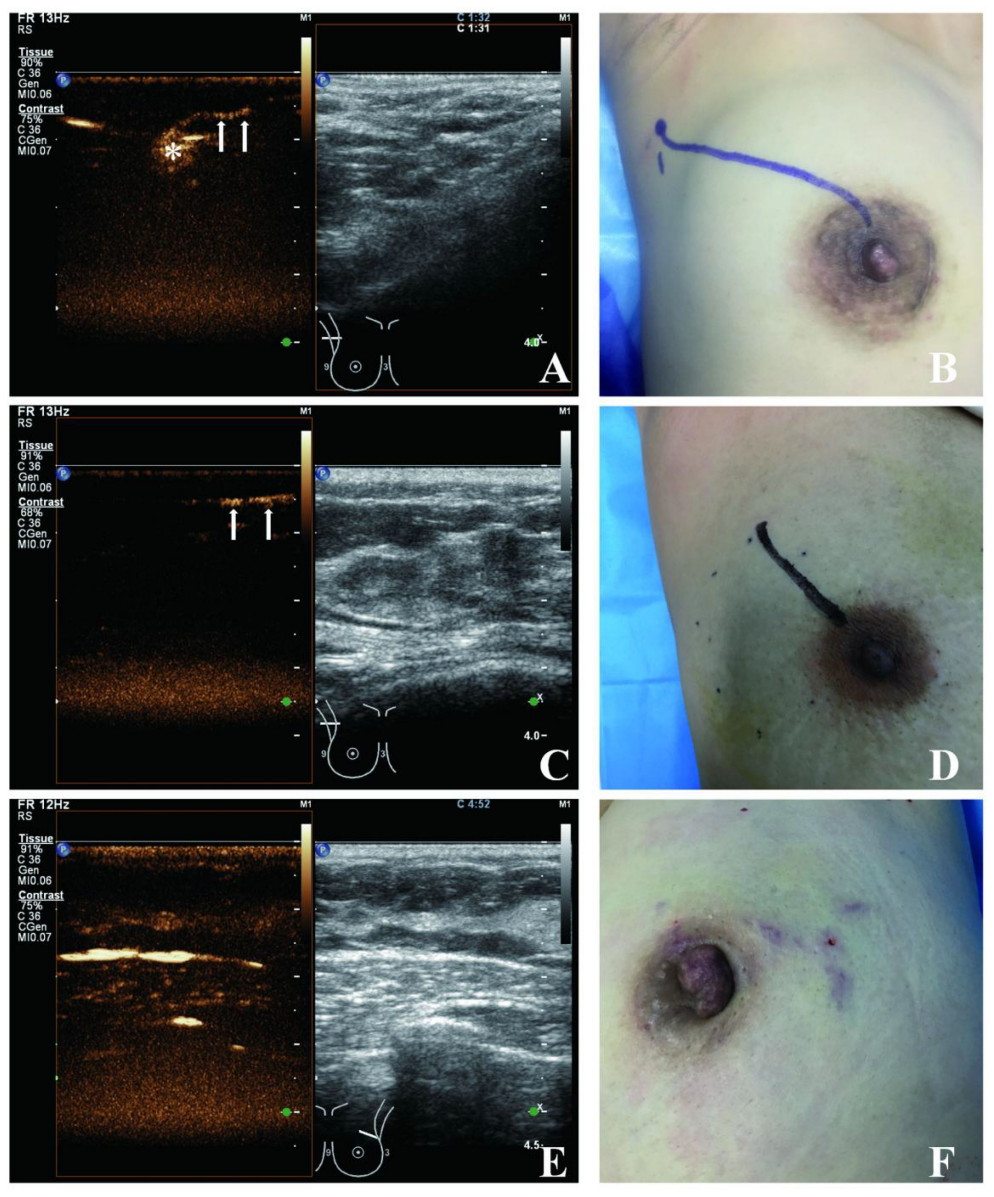
Examples of successful and unsuccessful CEUS. **(A, B)** Successful identification of SLN and SLC by CEUS. **(C, D)** Identification of SLC only not SLN by CEUS. **(E, F)** Failure of CEUS to identify SLN and SLC. *SLN, arrow: SLC.

Image acquisition was performed using a Philips iU Elite and a GE logic-E9 diagnostic ultrasound machine equipped with CEUS display system. CEUS were performed using L9-3 (Philips) and 9L (GE) linear array probes, with mechanical index (MI), 0.07 (Philips) and 0.16 (GE). SonoVue (Bracco spa, Milan, Italy) was used as the ultrasound contrast agent, and a bottle of SonoVue was prepared with 5 ml of saline. The contrast agent was injected intradermally and subcutaneously alternately, in which the four-point method was standardized to inject 1 ml at each point around the areola in the 3, 6, 9, and 12 o’clock directions, and the two-point method was standardized to inject 2 ml at each point in the direction of the upper and outer areola. A senior doctor and a junior doctor, both of whom had undergone standardized training in CEUS imaging of SLN, performed the procedure in this manner. The tumor location was depicted using a four-quadrant method (6). CEUS observation was performed 30–60 seconds after massaging the injection site after contrast agent injection. Successful CEUS imaging was defined as the continuous tracking of sentinel lymphatic channel (SLC) (6) from the periareolar region to the identification of SLN enhancement (**Figures 1A, B**). At 6 minutes after contrast agent injection, CEUS imaging was considered unsuccessful if only SLC enhancement was observed without SLN enhancement (**Figures 1C, D**), or if neither SLC nor SLN enhancement was detected (**Figures 1E, F**).

### 2.3 Surgery

In our study, the surgical retrieval of SLNs served as the gold standard for SLN diagnosis. During surgery, a standard blue staining method was employed for SLNB (6, 7), and if intraoperative frozen pathology indicated metastasis, the procedure was escalated to a standard axillary lymph node dissection (ALND). All SLNs removed intraoperatively were meticulously documented and compared with the SLNs identified by CEUS preoperatively to assess the accuracy of the CEUS technique against the surgical gold standard. In this study, only SLNs jointly identified by both preoperative CEUS and intraoperative methylene blue were included for analysis, ensuring the consistency between CEUS-identified SLNs and surgically removed ones. When CEUS imaging failed, intraoperative identification of SLNs was used as a reference.

### 2.4 Statistical analysis

Data processing and analysis were performed using R version 4.4.0. An internal training set and a validation set were randomly established at a ratio of 7:3. Non-normal continuous variables were described statistically using the median (P25, P75), differences between groups were analyzed using Mann-Whitney U test, and categorical variables were analyzed using the chi-square test or Fisher’s exact probability method. Binary logistic regression was used to analyze all influencing factors using the bidirectional variable selection.The performance of the model was assessed using the receiver operating characteristic (ROC) curve and its area under the curve (AUC), along with accuracy, sensitivity, specificity, positive predictive value (PPV), and negative predictive value (NPV). A 10-fold cross-validation method was used for internal model validation, and calibration curve analysis (CCA) was employed to evaluate the consistency between predicted probabilities and actual probabilities. The Hosmer-Lemeshow goodness-of-fit test was utilized to assess model calibration. Decision curve analysis (DCA) was performed to quantify the clinical net benefit. A nomogram was developed for risk prediction assessment. A two-tailed test with P<0.05 was considered statistically significant.

## 3 Results

### 3.1 Patient characteristics

**Figure 2:**
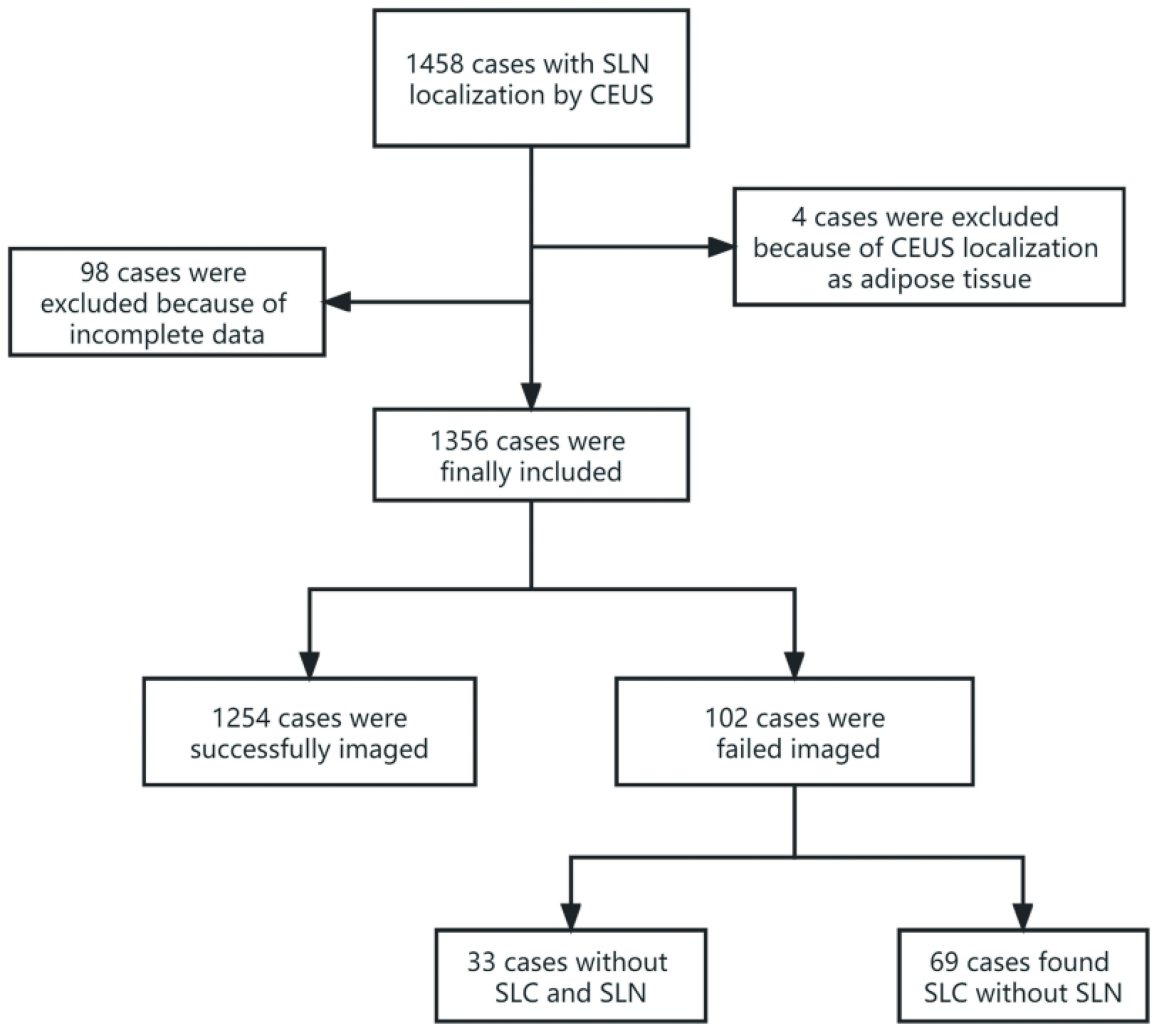
Study inclusion flowchart.

A total of 1356 cases were included in the study (**Figure 2**), with an age range of 21 to 88 years, of which 92.48% (1254/1356) had successful imaging, and 7.52% (102/1356) had failed imaging. In the successful group, a total of 2532 SLNs and 2227 non-SLNs were found, of which 217 cases had lymph node metastases, including 370 SLNs metastases and 207 non-SLNs metastases. In the failed group, 33 cases showed no enhancement of SLC and SLN, and 69 cases showed enhancement of SLC but no enhancement of SLN. The basic characteristics of the patients were shown in **Table 1**. The baseline characteristics of the training and validation sets were displayed in **Table 2**. There were no statistically significant differences in all characteristic indicators between the two groups (P > 0.05).

**Table 1:** Baseline characteristics of the patients.

Variables	Total (n = 1356)	Successful in CEUS (n = 1254)	Failed in CEUS (n = 102)	Statistic	*P*
Age(y), M (Q_1_, Q_3_)	49.00 (43.00, 57.00)	49.00 (43.00, 56.00)	52.00 (46.00, 60.75)	Z=-1.91	0.056
ER%, M (Q_1_, Q_3_)	80.00 (0.75, 90.00)	80.00 (0.00, 90.00)	80.00 (8.75, 90.00)	Z=-0.57	0.568
PR%, M (Q_1_, Q_3_)	20.00 (0.00, 80.00)	20.00 (0.00, 80.00)	30.00 (0.00, 80.00)	Z=-1.24	0.214
Ki67%, M (Q_1_, Q_3_)	20.00 (10.00, 30.00)	20.00 (10.00, 30.00)	20.00 (11.25, 40.00)	Z=-2.32	0.020
Maximum tumour diameter(mm), M (Q_1_, Q_3_)	20.00 (15.00, 30.00)	20.00 (15.00, 30.00)	22.00 (17.00, 35.00)	Z=-2.16	0.031
HER-2, n(%)				χ²=3.32	0.068
Negative	958 (70.65)	894 (71.29)	64 (62.75)		
Positive	398 (29.35)	360 (28.71)	38 (37.25)		
Operator, n(%)				χ²=0.53	0.465
Junior doctor	791 (58.33)	728 (58.05)	63 (61.76)		
Senior doctor	565 (41.67)	526 (41.95)	39 (38.24)		
Equipment, n(%)				χ²=0.05	0.823
GE LOGIQ E9	38 (2.80)	36 (2.87)	2 (1.96)		
Philip IU Elite	1318 (97.20)	1218 (97.13)	100 (98.04)		
Number of CEUS injection points, n(%)				χ²=3.43	0.064
Four	237 (17.48)	226 (18.02)	11 (10.78)		
Two	1119 (82.52)	1028 (81.98)	91 (89.22)		
Left or right, n(%)				χ²=0.28	0.600
Left	672 (49.56)	624 (49.76)	48 (47.06)		
Right	684 (50.44)	630 (50.24)	54 (52.94)		
Quadrant location of tumor, n(%)				χ²=4.81	0.028
In the outer upper quadrant	514 (37.91)	465 (37.08)	49 (48.04)		
In the other quadrant	842 (62.09)	789 (62.92)	53 (51.96)		
Metastasis of SLN, n(%)				χ²=32.62	<.001
No	1052 (77.58)	996 (79.43)	56 (54.90)		
Yes	304 (22.42)	258 (20.57)	46 (45.10)		
History of previous breast surgery, n(%)				χ²=4.66	0.031
No	1246 (91.89)	1158 (92.34)	88 (86.27)		
Yes	110 (8.11)	96 (7.66)	14 (13.73)		
History of neoadjuvant therapy, n(%)				χ²=48.80	<.001
No	1238 (91.30)	1164 (92.82)	74 (72.55)		
Yes	118 (8.70)	90 (7.18)	28 (27.45)		
Type of pathological, n(%)				χ²=1.93	0.380
DCIS	165 (12.17)	157 (12.52)	8 (7.84)		
IDC	1024 (75.52)	943 (75.20)	81 (79.41)		
Others	167 (12.32)	154 (12.28)	13 (12.75)		
Grade of histopathology, n(%)				χ²=5.97	0.113
1	103 (7.60)	94 (7.50)	9 (8.82)		
2	724 (53.39)	680 (54.23)	44 (43.14)		
3	220 (16.22)	203 (16.19)	17 (16.67)		
X*	309 (22.79)	277 (22.09)	32 (31.37)		
Type of molecular, n(%)				χ²=7.93	0.094
Luminal A	275 (20.28)	261 (20.81)	14 (13.73)		
Luminal B HER2 (-)	511 (37.68)	469 (37.40)	42 (41.18)		
Luminal B HER2(+)	205 (15.12)	183 (14.59)	22 (21.57)		
HER2 over-expression	193 (14.23)	177 (14.11)	16 (15.69)		
Basal-like	172 (12.68)	164 (13.08)	8 (7.84)		
Clinical prognostic stage, n(%)				χ²=62.47	<.001
≤IIb	1295 (95.50)	1214 (96.81)	81 (79.41)		
>IIb	61 (4.50)	40 (3.19)	21 (20.59)		

Z: Mann-Whitney test, χ²: Chi-square test; M: Median, Q_1_: 1st Quartile, Q_3_: 3rd Quartile. *Can’t be graded.

**Table 2:** Balance test of the training set and validation set.

Variables	Total (n = 1356)	Validation set (n = 407)	Training set (n = 949)	Statistic	*P*
Failed in CEUS, n(%)				χ²=0.10	0.756
No	1254 (92.48)	375 (92.14)	879 (92.62)		
Yes	102 (7.52)	32 (7.86)	70 (7.38)		
Age(y), M (Q_1_, Q_3_)	49.00 (43.00, 57.00)	50.00 (44.00, 56.00)	49.00 (43.00, 57.00)	Z=-0.16	0.873
ER%, M (Q_1_, Q_3_)	80.00 (0.75, 90.00)	80.00 (1.00, 90.00)	80.00 (0.00, 90.00)	Z=-0.26	0.797
PR%, M (Q_1_, Q_3_)	20.00 (0.00, 80.00)	20.00 (0.00, 80.00)	20.00 (0.00, 80.00)	Z=-0.68	0.496
Ki67%, M (Q_1_, Q_3_)	20.00 (10.00, 30.00)	20.00 (10.00, 30.00)	20.00 (10.00, 30.00)	Z=-1.34	0.18
Maximum tumour diameter(mm), M (Q_1_, Q_3_)	20.00 (15.00, 30.00)	20.00 (15.00, 28.00)	20.00 (15.00, 30.00)	Z=-0.41	0.685
HER-2, n(%)				χ²=0.71	0.401
Negative	958 (70.65)	294 (72.24)	664 (69.97)		
Positive	398 (29.35)	113 (27.76)	285 (30.03)		
Operator, n(%)				χ²=0.17	0.681
Junior doctor	791 (58.33)	234 (57.49)	557 (58.69)		
Senior doctor	565 (41.67)	173 (42.51)	392 (41.31)		
Equipment, n(%)				χ²=0.75	0.388
GE LOGIQ E9	38 (2.80)	9 (2.21)	29 (3.06)		
Philip IU Elite	1318 (97.20)	398 (97.79)	920 (96.94)		
Number of CEUS injection points, n(%)				χ²=0.20	0.655
Four	237 (17.48)	74 (18.18)	163 (17.18)		
Two	1119 (82.52)	333 (81.82)	786 (82.82)		
Left or right, n(%)				χ²=1.21	0.27
Left	672 (49.56)	211 (51.84)	461 (48.58)		
Right	684 (50.44)	196 (48.16)	488 (51.42)		
Quadrant location of tumor, n(%)				χ²=0.08	0.781
In the outer upper quadrant	842 (62.09)	255 (62.65)	587 (61.85)		
In the other quadrant	514 (37.91)	152 (37.35)	362 (38.15)		
Metastasis of SLN, n(%)				χ²=2.79	0.095
No	1052 (77.58)	304 (74.69)	748 (78.82)		
Yes	304 (22.42)	103 (25.31)	201 (21.18)		
History of previous breast surgery, n(%)				χ²=0.75	0.387
No	1246 (91.89)	370 (90.91)	876 (92.31)		
Yes	110 (8.11)	37 (9.09)	73 (7.69)		
History of neoadjuvant therapy, n(%)				χ²=0.57	0.451
No	1238 (91.30)	368 (90.42)	870 (91.68)		
Yes	118 (8.70)	39 (9.58)	79 (8.32)		
Type of pathological, n(%)				χ²=0.08	0.961
DCIS	165 (12.17)	48 (11.79)	117 (12.33)		
IDC	1024 (75.52)	309 (75.92)	715 (75.34)		
Others	167 (12.32)	50 (12.29)	117 (12.33)		
Grade of histopathology, n(%)				χ²=2.59	0.46
1	103 (7.60)	25 (6.14)	78 (8.22)		
2	724 (53.39)	227 (55.77)	497 (52.37)		
3	220 (16.22)	62 (15.23)	158 (16.65)		
X*	309 (22.79)	93 (22.85)	216 (22.76)		
Type of molecular, n(%)				χ²=2.62	0.623
Luminal A	275 (20.28)	89 (21.87)	186 (19.60)		
Luminal B HER2 (–)	511 (37.68)	159 (39.07)	352 (37.09)		
Luminal B HER2(+)	205 (15.12)	56 (13.76)	149 (15.70)		
HER2 over-expression	193 (14.23)	57 (14.00)	136 (14.33)		
Basal-like	172 (12.68)	46 (11.30)	126 (13.28)		
Clinical prognostic stage, n(%)				χ²=0.59	0.442
≤IIb	1295 (95.50)	386 (94.84)	909 (95.79)		
>IIb	61 (4.50)	21 (5.16)	40 (4.21)		

Z: Mann-Whitney test, χ²: Chi-square test; M: Median, Q_1_: 1st Quartile, Q_3_: 3rd Quartile. *Can’t be graded.

### 3.2 Logistic regression results

Univariate and multivariate logistic regression analysis of breast sentinel lymph node failure in CEUS were shown in **Table 3**. Logistic regression analysis showed that the quadrant location of tumor [(OR = 1.54, 95%CI: 1.01, 2.36)], the history of previous breast surgery [(OR = 3.05, 95%CI: 1.62, 5.72)], the metastasis of SLN [(OR = 2.68, 95%CI: 1.72, 4.15)], the history of neoadjuvant therapy [(OR = 2.90, 95% CI: 1.57, 5.37)], and the clinical prognostic stage [(OR = 3.50, 95% CI: 1.70, 7.22)] were the independent influencing factors of CEUS failure, with a statistically significant difference (P<0.05).

**Table 3:** Univariate and multivariate logistic regression analysis of breast SLN failure in CEUS.

Variables	Univariate					Multivariate				
	β	S.E	Z	*P*	OR (95%CI)	β	S.E	Z	*P*	OR (95%CI)
Clinical prognostic stage										
≤IIb					1.00 (Reference)					1.00 (Reference)
>IIb	2.06	0.29	7.04	<.001	7.87 (4.43 ~ 13.97)	1.25	0.37	3.39	<.001	3.50 (1.70 ~ 7.22)
History of neoadjuvant therapy										
No					1.00 (Reference)					1.00 (Reference)
Yes	1.59	0.25	6.42	<.001	4.89 (3.01 ~ 7.95)	1.06	0.31	3.39	<.001	2.90 (1.57 ~ 5.37)
Metastasis of SLN										
No					1.00 (Reference)					1.00 (Reference)
Yes	1.15	0.21	5.47	<.001	3.17 (2.10 ~ 4.79)	0.98	0.22	4.38	<.001	2.68 (1.72 ~ 4.15)
History of previous breast surgery										
No					1.00 (Reference)					1.00 (Reference)
Yes	0.65	0.31	2.13	0.034	1.92 (1.05 ~ 3.50)	1.11	0.32	3.46	<.001	3.05 (1.62 ~ 5.72)
Quadrant location of tumor										
In the other quadrant					1.00 (Reference)					1.00 (Reference)
In the outer upper quadrant	0.45	0.21	2.18	0.029	1.57 (1.05 ~ 2.35)	0.43	0.22	2.00	0.046	1.54 (1.01 ~ 2.36)
Ki-67%	0.01	0.00	2.36	0.018	1.01 (1.01 ~ 1.02)					
Maximum tumour diameter	0.02	0.01	3.17	0.002	1.02 (1.01 ~ 1.03)					
Type of molecular										
Luminal A					1.00 (Reference)					
Luminal B HER2(-)	0.51	0.32	1.61	0.107	1.67 (0.89 ~ 3.11)					
Luminal B HER2(+)	0.81	0.36	2.27	0.023	2.24 (1.12 ~ 4.50)					
HER2 over-expression	0.52	0.38	1.38	0.168	1.69 (0.80 ~ 3.54)					
Basal-like	-0.09	0.45	-0.21	0.834	0.91 (0.37 ~ 2.22)					
ER%	0.00	0.00	0.91	0.365	1.00 (1.00 ~ 1.01)					
Age	0.01	0.01	1.56	0.118	1.01 (1.00 ~ 1.03)					
PR%	0.00	0.00	1.08	0.282	1.00 (1.00 ~ 1.01)					
Type of pathological										
DCIS					1.00 (Reference)					
IDC	0.52	0.38	1.37	0.170	1.69 (0.80 ~ 3.55)					
Others	0.50	0.46	1.09	0.276	1.66 (0.67 ~ 4.11)					
Grade of histopathology										
1					1.00 (Reference)					
2	-0.39	0.38	-1.03	0.305	0.68 (0.32 ~ 1.43)					
3	-0.13	0.43	-0.31	0.756	0.87 (0.38 ~ 2.03)					
X*	0.19	0.40	0.47	0.635	1.21 (0.56 ~ 2.62)					
HER-2										
Negative					1.00 (Reference)					
Positive	0.39	0.21	1.81	0.070	1.47 (0.97 ~ 2.24)					
Operator										
Junior doctor					1.00 (Reference)					
Senior doctor	-0.15	0.21	-0.73	0.465	0.86 (0.57 ~ 1.30)					
Equipment										
GE LOGIQ E9					1.00 (Reference)					
Philip IU Elite	0.39	0.73	0.53	0.595	1.48 (0.35 ~ 6.23)					
Number of CEUS injection points										
Two					1.00 (Reference)					
Four	0.60	0.33	1.83	0.068	1.82 (0.96 ~ 3.46)					
Left or right										
Left					1.00 (Reference)					
Right	0.11	0.21	0.52	0.600	1.11 (0.74 ~ 1.67)					

OR, Odds Ratio; CI, Confidence Interval. *Can’t be graded.

### 3.3 Model evaluation and validation

**Figure 3:**
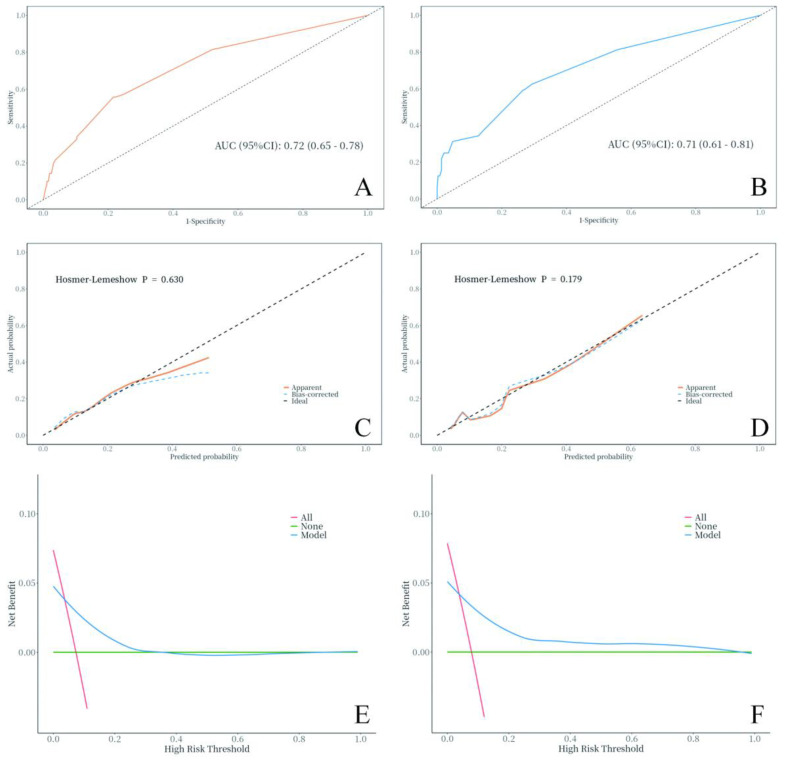
Model evaluation of the training set and validation set. **(A)** Training set ROC curve. **(B)** Validation set ROC curve. **(C)** Training set CCA. **(D)** Validation set CCA. **(E)** Training set DCA. **(F)** Validation set DCA.

For the training set, the AUC was 0.72 (95%CI: 0.65-0.78) (**Figure 3A**), accuracy was 0.77 (95%CI: 0.74-0.79), sensitivity was 0.78 (95%CI: 0.76-0.81), specificity was 0.56 (95%CI: 0.44-0.67), PPV was 0.96 (95%CI: 0.94-0.97), NPV was 0.17 (95%CI: 0.12-0.22), and the cut-off value was 0.092. For the validation set, the AUC was 0.71 (95%CI: 0.61-0.81) (**Figure 3B**), accuracy was 0.72 (95%CI: 0.68-0.77), sensitivity was 0.73 (95%CI: 0.69-0.78), specificity was 0.59 (95%CI: 0.42-0.76), PPV was 0.95 (95%CI: 0.93-0.98), NPV was 0.16 (95%CI: 0.09-0.23), with the cut-off value also being 0.092. The calibration curves of the training set (Hosmer-Lemeshow test p=0.630) and validation set (Hosmer-Lemeshow test p=0.179) indicated no significant deviation between the predicted risk of CEUS failure and the actual observed failure rate (**Figures 3C, D**). The decision curves of the training set and validation set suggested that clinical decisions guided by the model can provide patients with additional net benefits (**Figures 3E, F**).

### 3.4 Nomogram for predicting the failure of CEUS in imaging SLN

**Figure 4:**
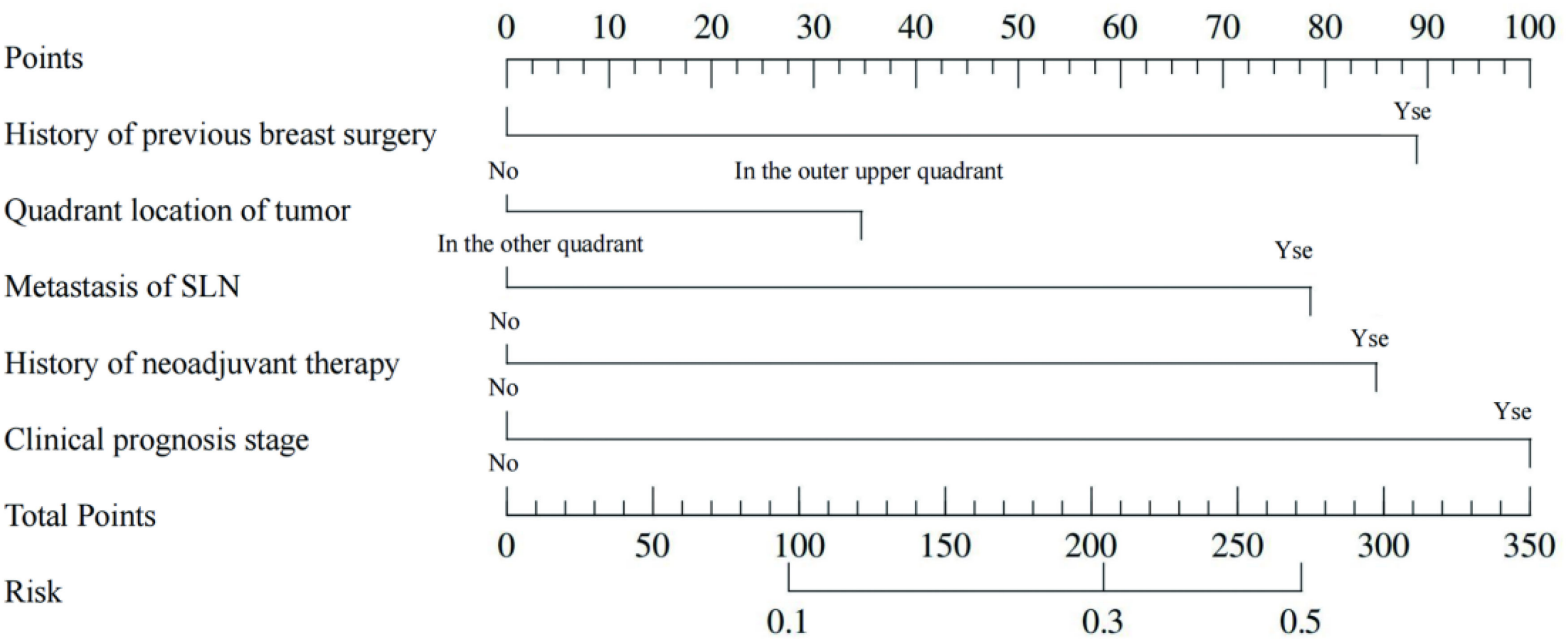
Nomogram for the risk of the failure of CEUS in imaging SLN.

This study developed a nomogram to predict the risk of CEUS failure in imaging SLN (**Figure 4**), incorporating the following predictive variables: tumor quadrant location, history of previous breast surgery, SLN metastasis status, history of neoadjuvant therapy, and clinical prognostic stage.

## 4 Discussion

CEUS is a safe, effective and radiation-free method of SLN imaging, but there are still some patients who are unable to image the SLN. This study retrospectively analyzed the results of CEUS in 1356 patients, and found the influencing factors of the failure of CEUS imaging, which has a good clinical application value. The results of this study can optimize the use of medical resources, improve the quality of medical care, and reduce the misunderstanding of patients.

### 4.1 Classification and interpretation of factors influencing CEUS failure

The factors leading to CEUS imaging failure can be categorized into metastasis-related factors and non-metastasis-related factors, which carry distinct clinical implications and require differentiated interpretation.

#### 4.1.1 Metastasis-related factors for CEUS failure

SLN with complete metastasis is destroyed by tumor cells, resulting in the absence of normal lymphoid tissue, so it cannot be recognized by translymphatic CEUS. Moreover, metastatic tumor cells may also cause abnormalities in the lymphatic system, resulting in the inability of CEUS imaging (9). In addition, lymph node metastasis may be accompanied by inflammatory reactions and edema in local tissues (10, 11), further interfering with the CEUS imaging. Clinical prognostic staging is an important indicator for evaluating the severity and prognosis of malignant tumors. For patients with stages greater than IIb, the burden of lymph node metastasis is higher, the primary tumor is larger, and it frequently invades surrounding tissues or organs, or distant metastases may already be present (12). Such patients are usually due to the invasion and destruction of lymphatic vessels by the primary tumor, or the tumor directly compresses lymphatic vessels, resulting in lymphatic obstruction. In addition, as mentioned above, the high metastasis load on lymphatic vessels is also an important factor leading to the failure of CEUS imaging. Non-enhancement of SLN caused by metastasis carries direct diagnostic value—it may indicate SLN involvement, suggesting the need for further pathological confirmation and adjustment of surgical plans (e.g., axillary lymph node dissection instead of SLN biopsy).

#### 4.1.2 Non-metastasis-related factors for CEUS failure

The lymphatic drainage of the breast typically originates from the areola and follows a path along the outer upper quadrant to the axillary region (6). When a tumor is located in the outer upper quadrant, its compression or invasion of the lymphatic vessels may impair lymphatic drainage, thereby affecting the ability of the CEUS agent to reach the SLN. Patients with a history of previous breast surgery could have scar tissue or anatomical changes in the surgical area (11, 13). Such factors may affect the absorption and drainage of ultrasound contrast agent, and thus affect the imaging quality of CEUS. Neoadjuvant therapy such as chemotherapy and radiotherapy, play an important role in the treatment of malignant tumors (14), but these therapies may also have an impact on CEUS imaging. Chemotherapy can damage lymphatic endothelial cells, affect the permeability and integrity of lymphatic vessels, and lead to edema (15). This impairment of lymphatic function will interfere with the normal perfusion and retention of contrast agent in lymphatic vessels, thus affecting the imaging of CEUS. Radiotherapy can lead to local tissue fibrosis and lymphatic vessel damage, also affects CEUS imaging (16). Non-enhancement caused by non-metastatic factors does not indicate SLN involvement but reflects technical limitations of CEUS in specific patient groups. For these patients, alternative SLN localization methods (e.g., radioactive colloid or blue dye) should be considered preoperatively to avoid false-negative imaging results.

The main reasons for the failure of CEUS imaging in this study are that these factors can directly or indirectly affect the perfusion or distribution of contrast agent, thus interfering with the CEUS imaging.

### 4.2 Analysis of variables with inconsistent significance in univariate and multivariate analysis

In this study, we found that some variables showed statistical significance in the univariate analysis but lost their significance in the multivariate analysis. This finding suggests that the interaction of multiple factors needs to be considered comprehensively when assessing predictors of CEUS imaging failure in breast cancer patients. For example, both Ki-67% and maximum tumour diameter were significantly associated with CEUS imaging failure in univariate analysis, but their effect was attenuated in the multivariate models. This attenuation may occur because the impact of these variables is obscured by other more influential predictors (e.g., clinical prognostic stage and metastasis burden) or because they are associated with various confounding factors (e.g., Ki-67% correlates with tumor invasiveness, which is already reflected in clinical staging).

The results of this study found that the equipment of CEUS, the operator of CEUS, the number of contrast agent injection points, and the tumor of left or right were not influencing factors in the failure of CEUS for SLN imaging. Previous research has demonstrated that doctors can master the CEUS technique after a cumulative total of 25 cases (3). This was supported by the results from our study, which showed no significant difference in the failure rate between junior and senior doctors, suggesting that CEUS imaging of SLN was independent of the operator and the equipment used to perform the procedure. Instead, the influencing factors were more likely to be related to the progression of the breast cancer and the patient’s own condition.

### 4.3 Exploration of non-significant pathological and immunohistochemical factors

Although our study found that pathological results, including the type of pathology and histopathological grade, as well as immunohistochemical features such as HER-2 status, ER%, and PR%, did not statistically significantly affect the failure rate of CEUS imaging, several potential explanations for this lack of significance should be considered. Pathological and molecular subtypes may not act independently but interact with other predictors (e.g., tumor location or neoadjuvant therapy). For example, HER-2-positive tumors may have higher invasiveness, but this effect may be masked by the more dominant influence of clinical staging (>IIb) on lymphatic obstruction. As clinical prognostic staging is integrated with pathological and molecular features, the impact of these factors may already be encapsulated in the staging variable, leading to their non-significance in multivariate models. In particular, the clinical prognostic stage, which is determined by these pathological results and molecular tumor typing, has been shown to significantly influence CEUS imaging outcomes. Histopathological grades reflect the invasiveness and biological behavior of tumors, which can indirectly affect the tumor microenvironment, including the structure and function of lymphatic vessels. These microenvironmental changes could, in turn, affect the success of CEUS imaging. For example, highly invasive tumors may trigger local tissue reactions that can subsequently affect the patency of lymphatic vessels and the distribution of contrast agents. Immunohistochemical features such as HER-2, ER%, and PR% are crucial for classifying breast cancer subtypes. These features not only guide treatment decisions but may also correlate with the tumor’s invasiveness into surrounding tissues and the integrity of lymphatic vessels. These pathological and immunohistochemical characteristics could interact with other known influencing factors, such as tumor location and metastatic status. While such interactions might be challenging to quantify in statistical models, they could significantly affect CEUS imaging outcomes in clinical practice. Consequently, even if these factors do not demonstrate an independent effect in statistical analyses, it is advisable to consider their implications and their relationship with clinical prognostic staging when interpreting CEUS imaging results. Future research should investigate the combined and interactive effects of these factors on the success of CEUS imaging.

### 4.4 Specific clinical applications of the study findings

Understanding the risk factors for the failure of CEUS SLN imaging is of great significance for clinical practice (17). By identifying patients at high risk for CEUS imaging failure, we can implement targeted strategies to optimize preoperative SLN localization. Firstly, patients with high-risk features (clinical stage >IIb, outer upper quadrant tumors, history of breast surgery, or neoadjuvant therapy) should be pre-identified. For these patients, CEUS can be used as a preliminary assessment, but alternative localization methods (e.g., radioactive colloid injection) should be prepared in advance to avoid intraoperative SLN localization failure. Secondly, for patients with non-metastatic risk factors (e.g., post-surgical scarring), adjusting the contrast agent injection site (avoiding scar tissue) or increasing the injection volume appropriately may improve imaging success, though this requires further validation. Thirdly, when CEUS shows “no SLN enhancement” in patients with low metastasis risk (e.g., stage ≤IIb, no prior surgery), clinicians should prioritize non-metastatic causes and consider repeating CEUS or switching to other modalities, rather than directly opting for axillary dissection.

Beyond preoperative localization, CEUS, as a non-invasive detection method, offers real-time, radiation-free, high-resolution imaging, which is essential for accurate preoperative localization of SLN (18). Some researchers have started using CEUS as a non-invasive method to detect ALN metastasis (19). The status of ALNs serves as a critical prognostic indicator for breast cancer patients, making accurate imaging of ALNs essential for crafting personalized treatment plans and forecasting patient outcomes. From a public health perspective, enhancing the success rate of CEUS imaging can decrease the dependency on invasive surgical procedures (e.g., unnecessary axillary dissection), reduce healthcare costs, and improve patient satisfaction. Consequently, thorough research into and comprehension of the risk factors for CEUS imaging failure can not only advance CEUS technology but also offer robust support for the clinical management of breast cancer.

### 4.5 Limitations

There were also some shortcomings in this study. Up to now, no other scholars have reported the specific reasons for the failure of CEUS for SLN imaging, so there was a lack of research results for direct comparison in this field. Second, the data in this study were obtained from a single medical center, and there may be differences in patient population characteristics, equipment performance, operating procedures, and healthcare personnel experience between different medical centers, which may affect the CEUS imaging. Finally, this study mainly focused on SonoVue contrast agent, while other types of contrast agents, such as Sonazoid, were not investigated. There were differences in the physical and chemical properties of different contrast agents, which may affect the distribution pattern, clearance rate, and ultimately the imaging effect of the contrast agents *in vivo*. Therefore, the conclusions and results of this study may not be fully applicable to other types of contrast agents without further validation.

## 5 Conclusions

The quadrant location of tumor, the history of previous breast surgery, the metastasis of SLN, the clinical prognostic stage and the history of previous neoadjuvant therapy were the independent influencing factors for the failure of CEUS for SLN imaging in breast cancer.

## Data Availability

The raw data supporting the conclusions of this article will be made available by the authors, without undue reservation.
